# Microduplication of 15q13.3 and Microdeletion of 18q21.32 in a Patient with Moyamoya Syndrome

**DOI:** 10.3390/ijms19113675

**Published:** 2018-11-20

**Authors:** Sciacca Francesca Luisa, Rizzo Ambra, Bedini Gloria, Capone Fioravante, Di Lazzaro Vincenzo, Nava Sara, Acerbi Francesco, Rossi Sebastiano Davide, Binelli Simona, Faragò Giuseppe, Gioppo Andrea, Grisoli Marina, Bruzzone Maria Grazia, Ferroli Paolo, Pantaleoni Chiara, Caputi Luigi, Vela Gomez Jesus, Parati Eugenio Agostino, Bersano Anna

**Affiliations:** 1 Dipartimento di Diagnostica e Tecnologia Applicata, Fondazione IRCCS Istituto Neurologico Carlo Besta, 20133 Milan, Italy; francesca.sciacca@istituto-besta.it (S.F.L.); ambra.rizzo@istituto-besta.it (R.A.); 2 Laboratory of Cellular Neurobiology, Fondazione IRCCS Istituto Neurologico Carlo Besta, 20133 Milan, Italy; gloriabedini85@gmail.com (B.G.); sara.nava@istituto-besta.it (N.S.); 3 Unit of Neurology, Neurophysiology, Neurobiology, Department of Medicine, Università Campus Bio-Medico di Roma, 00128 Rome, Italy; f.capone@unicampus.it (C.F.); v.dilazzaro@unicampus.it (D.L.V.); 4 Neurosurgical Unit, Fondazione IRCCS Istituto Neurologico Carlo Besta, 20133 Milan, Italy; francesco.acerbi@istituto-besta.it (A.F.); paolo.ferroli@istituto-besta.it (F.P.); 5 Neurophysiopathology Department and Epilepsy Centre, Fondazione IRCCS Istituto Neurologico Carlo Besta, 20133 Milan, Italy; davide.rossi@istituto-besta.it (R.S.D.); simona.binelli@istituto-besta.it (B.S.); 6 Neuroradiological Unit, Fondazione IRCCS Istituto Neurologico Carlo Besta, 20133 Milan, Italy; giuseppe.farago@istituto-besta.it (F.G.); andrea.gioppo@istituto-besta.it (G.A.); marina.grisoli@istituto-besta.it (G.M.); maria.bruzzone@istituto-besta.it (B.M.G.); 7 Developmental Neurology Division, Fondazione IRCCS Istituto Neurologico Carlo Besta, 20133 Milan, Italy; chiara.pantaleoni@istituto-besta.it; 8 Cerebrovascular Unit, Fondazione IRCCS Istituto Neurologico Carlo Besta, 20133 Milan, Italy; luigi.caputi@istituto-besta.it (C.L.); Jesus.VelaGomez@istituto-besta.it (V.G.J.); eugenio.parati@istituto-besta.it (P.E.A.)

**Keywords:** moyamoya, genetic, syndrome, 15q13.3 microduplication, 18q21.32 microdeletion

## Abstract

Moyamoya angiopathy (MA) is a cerebrovascular disease determining a progressive stenosis of the terminal part of the internal carotid arteries (ICAs) and their proximal branches and the compensatory development of abnormal “moyamoya” vessels. MA occurs as an isolated cerebral angiopathy (so-called moyamoya disease) or in association with various conditions (moyamoya syndromes) including several heritable conditions such as Down syndrome, neurofibromatosis type 1 and other genomic defects. Although the mechanism that links MA to these genetic syndromes is still unclear, it is believed that the involved genes may contribute to the disease susceptibility. Herein, we describe the case of a 43 years old woman with bilateral MA and peculiar facial characteristics, having a 484-kb microduplication of the chromosomal region 15q13.3 and a previously unreported 786 kb microdeletion in 18q21.32. This patient may have a newly-recognized genetic syndrome associated with MA. Although the relationship between these genetic variants and MA is unclear, our report would contribute to widening the genetic scenario of MA, in which not only genic mutation, but also genome unbalances are possible candidate susceptibility factors.

## 1. Introduction

Moyamoya angiopathy (MA) is a rare (prevalence close to 6/100,000 in Japan, around ten-times less in Europe) cerebrovascular disease characterized by a progressive stenosis of the terminal part of the supraclinoid internal carotid arteries (ICAs) and their proximal branches, resulting in a compensatory development of fragile basal collateral vessels (moyamoya vessels) [1,2]. This condition leads to a severe hemodynamic impairment, conditioning ischemic strokes and cerebral hemorrhages, which are the most important disease clinical features. MA is commonly classified in moyamoya disease (MMD) when MA is bilateral, which is the idiopathic disease form, or moyamoya syndrome (MMS), or pseudo-moyamoya, or quasi-moyamoya when MA is associated with a series of well-known acquired (i.e., head irradiation, autoimmune disorders) or constitutional (genomic unbalances or mutations) conditions. Several genetic syndromes such as sickle cell disease, neurofibromatosis type 1, and Down, Noonan, Costello, and Alagille syndromes have been associated with MA [3,4].

The etiology of MA is still unknown. Although the detection of increased cytokine and growth factor concentration would support the role of impaired angiogenic and arteriogenic mechanism, genetic factors have long been considered, given the high familial rate (10–15%) and the ethnic differences [4,5,6]. The inheritance pattern and the involved genes are mostly unknown. *RNF213* gene encoding for an enzyme with a Really Interesting New Gene (RING) finger and AAAA+ Adenosine TriPhosphate (ATP)ase domain is the only gene identified as conferring susceptibility to MA in Asian countries [7,8]. However, although missense mutations in this gene have been recently significantly associated with MA European patients, particularly in childhood-onset and familial cases [7,8], the causative role of this gene in Caucasians is still debated [9]. Recently, familial cases of MA associated with peculiar facial characteristics have been identified by copy number analysis, detecting microdeletion in Xq28 involving the *BRCC3* gene that encodes a 36-kDa ubiquitous deubiquitinating enzyme [10]. Risk of MA disease has been also attributed to mutations in *GUCY1A3* gene, encoding the major nitric oxide receptor in vascular smooth muscle cells (vSMCs) in achalasia cases [11]. Other sporadic syndromic cases of MA have been reported, as resumed in Table 1 [10,11,12,13,14,15,16,17,18,19,20,21,22,23,24,25,26,27,28,29,30,31,32,33]. However, these observations are not able to fully explain the pathogenesis of MA, which is believed to be much more complex. A mechanism including the role of multiple genes and angiogenic abnormalities, in which unknown triggering environmental factors (including infections, immune failure, and hemodynamic stress to specific vascular loci), has been postulated for starting the first pathological changes of the disease [5,6].

Herein, we describe the case of a 43 years old woman with bilateral MA and peculiar facial characteristics, belonging to the population of the GENetics of mOyaMoyA (GENOMA) project, screened for copy number imbalances as suggested by the syndromic phenotype, resulting in a 484-kb microduplication of the chromosomal region 15q13.3, previously associated with clinical phenotype having incomplete penetrance [34] and a previously unreported 786-kb microdeletion in 18q21.32. This patient may have a newly-recognized genetic syndrome associated with MA.

## 2. Case Report

**Figure 1:**
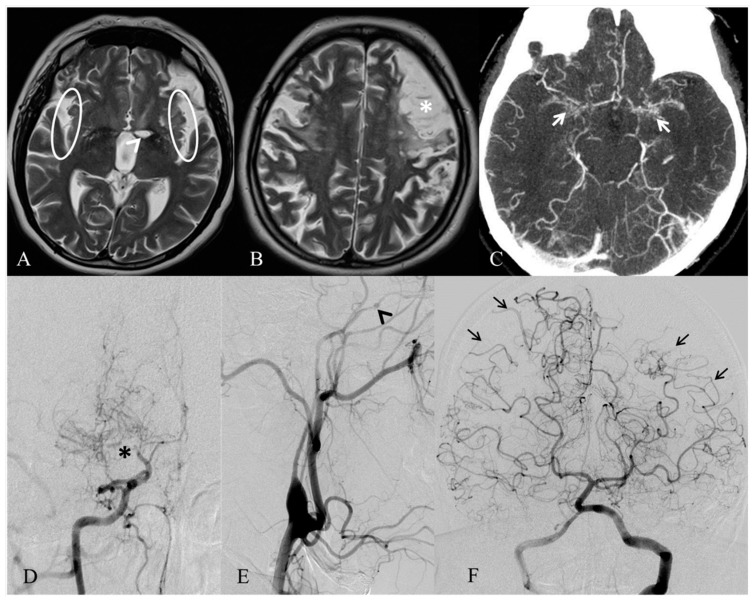
MRI and angiographic images of MA of our case. Axial T2-weighted MRI (**A**,**B**) exhibits only a few vascular voids in the Sylvian fissures (white circles), due to a reduced flow in both the middle cerebral arteries. A well-demarcated ischemic stroke in the left nucleo-capsular region (white arrowhead) and an infarct involving the left frontal lobe (white asterisk) are also noticeable. Axial angio-CT (**C**) shows proliferation and enlargement of the lenticulostriate arteries (white arrows) in the basal ganglia. Right and left common carotid arteries’ angiograms (**D**–**E**) demonstrate a distal stenosis of the right internal carotid artery (black asterisk), with development of prominent collateral vessels, giving the characteristic puff of smoke appearance of moyamoya disease, and an occlusion of the left internal carotid artery, distally to the ophthalmic artery (black arrowhead). Left vertebral artery angiogram (**F**) indicates leptomeningeal cortical anastomosis from both the posterior cerebral arteries to the parietal and temporal lobes (black arrows).

A 43 years old woman, born from non-consanguineous parents and with no documented developmental structural or mental defects, presented in February 2014 a progressive dysarthria, motor slowness, and changes of behavior, becoming listless and aggressive. In April 2014 she presented a sudden aphasia and right hemiparesis for which she was hospitalized elsewhere. The acute phase cerebral Computed Tomography (CT) and Magnetic Resonance Imaging (MRI) scan showed acute cortico-subcortical left fronto-parietal and left internal capsule acute lesions. Cerebral Computed Tomography Angiography (CTA) scan detected a bilateral stenosis of internal carotid arteries (ICAs), middle cerebral arteries (MCAs), and anterior cerebral arteries (ACAs). Coagulation and autoimmunity screening were normal, as well as electrocardiography (ECG) monitoring and echocardiography. Aspirin therapy was introduced, and the patient was addressed to a rehabilitation course. After an initial improvement of the right hemiparesis, her clinical status worsened, and she developed a vertical ophthalmoparesis and dystonic movements in the left arm. For these symptoms, a biochemistry examination was performed, which resulted in being normal, as well as an abdominal ultrasound examination showing liver steatosis. A new cerebral MRI did not show any changes and detected the previously observed left frontal and insular lesions, as well as new right frontal and parietal lesions. Cerebral MRA confirmed the severe stenosis of the ICAs, MCAs, and ACAs, mostly in the left side (Figure 1). Thus, a suspicion of moyamoya angiopathy was posed, and in June 2015, she was addressed to our center. The neurological evaluation, performed at that time, showed the presence of severe attention deficit, orofacial apraxia, dysarthria, slight left hemianopia, and right hemiparesis with bilateral coreoatetosic movements and Babinski sign. Peculiar characteristics were a long narrowing face with bifrontal prominence, a low frontal hairline, arched eyebrows, downslanting palpebral fissures, hypertelorism, broad nasal tip, upturned nares, and large and low-set ears. Digital subtraction angiography (DSA) revealed stenosis of the ICAs with apex occlusion in the right one and hypertrophic aspects of lenticulostriate arteries with leptomeningeal anastomosis from ophthalmic artery and frontal artery to provide blood flow to the frontal regions. Blood flow in the temporal, parietal, and occipital regions was supported by posterior cerebral artery. Electroencephalography revealed left temporal epileptic activity. Perfusion MRI showed a decreased relative cerebral blood flow (rCBF) and cerebral blood volume (rCBV) into ischemic areas, as well as a bilateral increased time to peak (rTTP) and mean transit time (rMTT). A diagnosis of MA was confirmed, and given the somatic peculiar characteristics and the cognitive impairment associated with the MA, the patient underwent an array-comparative genomic hybridization (aCGH), after informed consent.

In September 2016 and in May 2017, the patient underwent a direct (STA-MCA) and indirect revascularization (encephalo-duro-arterio-synangiosis (EDAS)) surgery in the left and right hemispheres, respectively. The post-surgical clinical course was regular, and the post-surgery DSA confirmed the by-pass perviety. A few months later, she presented focal seizures for which she performed a new electroencephalogram showing left parietotemporal sharp waves and started treatment with levetiracetam, with complete resolution of seizures. At the two-year follow-up evaluation, she did not refer to any other symptoms, and we did not detect any other signs of cerebral or systemic involvement.

## 3. Results

**Figure 2:**
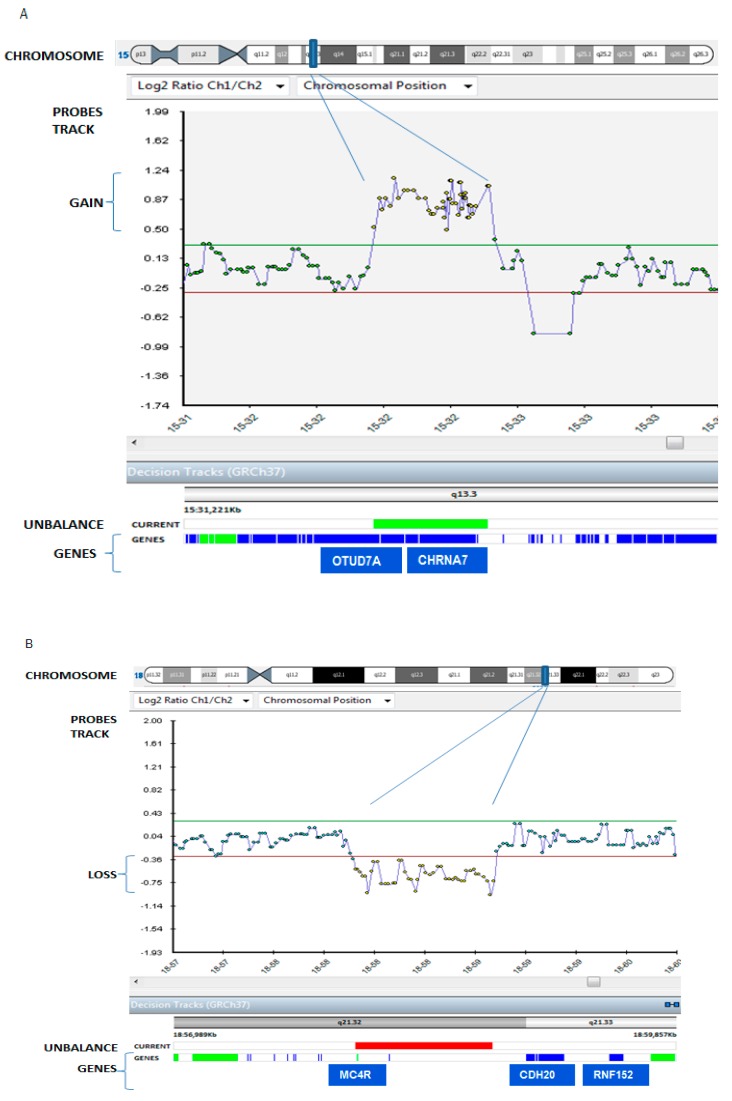
Patient’s array comparative genomic hybridization (aCGH) data result. The patient carried: (panel A) a 484 Kb microduplication in the chromosomal region 15q13.3 (the blue bar in the chromosome track), from 32065077 to 32539670 chromosome coordinate, zoomed in the probes track as indicated by purple lines, and (panel B) a 786 Kb microdeletion in the 18q21.32 region (the blue bar in the chromosome), from 58014483 to 58816361 chromosome coordinate, zoomed in the probes track as indicated by purple lines. Duplication and deletion are revealed by a disclosure of at least 3 probes from the +0.4 (duplication)/−0.6 (deletion range) of Log_2_ratio value in X axis (normal values are included in the range from +0.30, green line, to −0.30, red line; probed duplicated and deleted in the patient are yellow in the figure, while green dots are balanced probes. In the track “unbalance”, the patient’s duplication in panel A and the patient’s deletion in panel B are indicated with a green and red bars respectively. Genes are represented as blue strips in the genes truck, and genes associated with pathology are colored in green.

The aCGH analysis of the patient revealed a 484-kb microduplication of the chromosomal region 15q13.3 (from the 32065077–32539670 chromosome coordinate), involving the genes *OTUD7A*, coding for Ovarian Tumor Domain (OTU) deubiquitinase 7A, and *CHRNA7*, coding for neuronal nicotinic cholinergic receptor alpha polypeptide 7, and a 786-kb microdeletion in the 18q21.32 (from 58014483–58816361), involving the gene *MC4R*, coding for melanocortin 4 receptor, and a regulatory element lincRNA MIR122 (Figure 2).

Array-CGH data of the patient showed a 484-kb microduplication of the chromosomal region 15q13.3, from the 32065077–32539670 chromosome coordinate (Panel A), and a 786-kb microdeletion in the 18q21.32 region, from 58014483–58816361 (Panel B). Duplication and deletion were revealed by a disclosure of at least three probes from the −0.6 (deletion)/+0.4 (duplication) range of the Log_2_ ratio value in the X axis (probed duplicated, and deleted in the patient are yellow in the figure).

**Figure 3:**
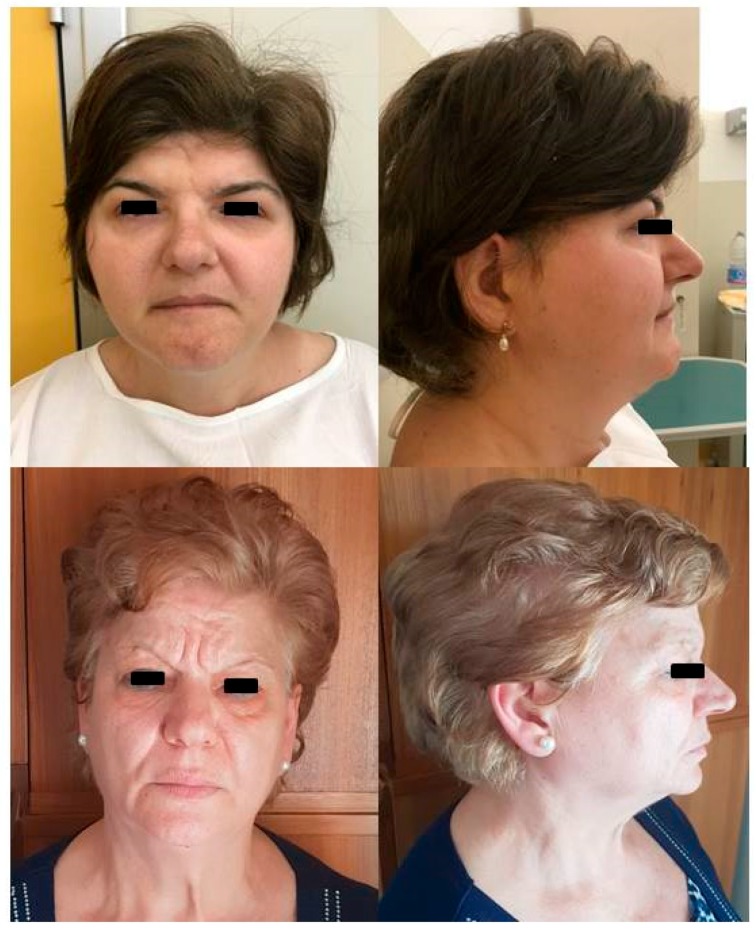
Proband and proband’s mother’s photos. Front and profile photos of the proband (upper panels) and her mother (lower panels). The proband shows peculiar characteristics such as a long narrowing face with bifrontal prominence, low frontal hairline, arched eyebrows, downslanting palpebral fissures, hypertelorism, broad nasal tip, upturned nares, and large and low-set ears. The mother is similar, but not having low frontal hairline and hypertelorism.

Both unbalances resulted by being inherited from the mother, who did not have neurovascular signs, but looked similar to the proband (Figure 3) and presenting less dysmorphic features than her daughter (low frontal hairline and hypertelorism were not present in the mother). The 15q13.3 microduplication was detected also in the healthy patient’s brother and sister. We did not perform the aCGH analysis on the 13 y.o patient’s son since he was underage. This unbalance is a common variant with a described low penetrance in neuropsychiatric disorders [34,35,36,37], and it has never been previously reported to be associated with vascular diseases. The microdeletion 18q21.32 was rarely observed and not reported in the databases of normal variants. The patient’s siblings did not have this unbalance and were not phenotypically similar to the patient. We analyzed the mRNA expression of *MC4R* and mir-122, located in the deleted region, and *RNF152*, which is located distal to the deletion, in position (59475296_59561480) of chromosome 18. mRNA expression of these three genes was detectable, but not significantly different in the patient compared to her relatives (parents, one brother, and one sister) and to the pool of healthy controls (data not shown).

## 4. Discussion

Herein, we describe the first MA patient having a microduplication of the chromosomal region 15q13.3 and a microdeletion in the 18q21.32. Our patient, other than the typical moyamoya neuroradiological features, has some peculiar facial characteristics such as a long narrowing face with bifrontal prominence, low frontal hairline, arched eyebrows, downslanting palpebral fissures, hypertelorism, broad nasal tip, upturned nares, and large and low-set ears. Facial dysmorphism, which commonly includes a combination of different facial features, is a well-known characteristic of many genetic syndromes and has been described in moyamoya syndromic cases [4,38]. The phenotypic presentation and the genomic unbalances observed in our patient supported the diagnosis of moyamoya syndrome, rather than moyamoya disease. Microduplications of 15q13.3 ranging from 350 kb–1.6 Mb involving *OTUD7A* and *CHRNA7* have been previously described, with various breakpoints within the 15q13.3 region. *OTUD7A* encodes for out deubiquitinase 7A, a protein expressed mainly in the central nervous system and having a supposed tumor suppressor role. *CHRNA7* encodes the α7 subunit of the neuronal nicotinic acetylcholine receptor (nAChR), which is composed of five subunits, arranged in a barrel-like configuration in the cell membrane. The subunits exist as 16 isoforms (α1–α10, β1–β4, δ, γ, and ε), which may be arranged in homomeric or heteromeric configurations. Each subunit contains four domains (M1–M4) in the cell membrane, with the M2 transmembrane domain of each subunit contributing to the channel of the receptor. Combinatorial association of various α- and β-subunits result in functionally-diverse nAChR subtypes that vary in ion permeability, open time, ligand affinity, and other functions [36,37,39]. Given the low estimation of penetrance, the pathogenicity of 15q13.3 duplication encompassing *OTUD7A* and *CHRNA7* has been associated with a highly variable intra- and inter-familial phenotype. Patients with such microduplication exhibit various clinical symptoms varying from asymptomatic or mild developmental delay and learning problems during childhood to mental retardation, autism, muscular hypotonia, and a variety of neuropsychiatric disorders, including seizures [34,35,37]. Although MA has never been described as an associated finding, we cannot completely exclude a possible role of such a microduplication in endothelial dysfunction, since in the endothelial cells, the α7 homomeric nAChR has been observed to act as a mediator of cholinergic angiogenesis and to be upregulated in in vitro and in vivo ischemic experimental conditions [39]. However, since this variant was present also in the proband’s healthy relatives, it is probably benign or has incomplete penetrance.

More interestingly, the patient carried also a microdeletion of 18q21.32, in the site of the *MC4R* (melanocortin-4 receptor) gene. Haploinsufficiency of the *MC4R* has been described as the most common heritable obesity syndrome in man and is responsible for 0.5–2.5% of all early onset morbid obesity [40]. Moreover, *MC4R* has been found to be expressed most heavily in the central nervous system, where it plays a critical role in energy balance [41]. *MC4R*-expressing neurons within the hypothalamus receive orexigenic and anorexigenic inputs from arcuate nucleus pro-opiomelanocortin POMC and agouti-related protein-expressing neurons AgRP projections and act to maintain energy homeostasis through modulation of both food intake and energy expenditure [42,43].

However, MC4R has been reported to be involved also in angiogenic balance, endothelial function, and blood pressure control. MC4R+/− obese pregnant rats were observed to have a pro-angiogenic phenotype with circulating VEGF greater that MC4R+/+ pregnant rats [42,43].

Notably, the deleted region included a non-coding microRNA (miRNA), mir-122, which is considered as a potential biomarker in acute liver failure. Because miRNAs are regulator of mRNA expression, mir-122 could influence the expression of genes located nearby: we noticed a gene of the ring finger protein (*RNF*) family, *RNF152*, close to the above-mentioned microdeletion region. RNF152 is an E3 ligase and is self-polyubiquitinated. It is localized in lysosomes and displays a pro-apoptosis activity when overexpressed [44]. Given the well-known association between *RNF213* and MA, we speculated on a possible role of this ring finger protein, *RNF152*, in the development of MA in our patient. Since this variant was not included in the deleted region, we hypothesized that deletion of a regulatory element, such as mir-122, may influence the expression of the flanking gene in a tissue-specific manner and with variable penetrance and expressivity. In fact, 18q21.32 microdeletion was carried also by the mother of our patient, who did not present MA. For this reason, we analyzed the expression of mir-122 and *RNF152* to evaluate a supposed mutual modulation. Unfortunately, the expression data did not show any differences in *RNF152* mRNA expression comparing our patient with relatives or controls.

Although we cannot exclude a different way of regulation or a tissue-dependent expression variability of mir-122, *MC4R* and *RNF152*, which could have a different behavior in the central nervous system, we believe that the microdeletion or microduplication might be primarily responsible for the disease susceptibility,. Other modifiers, genetic and environmental factors could ultimately trigger the phenotypic presentation, explaining the variable phenotypes within the patient and her mother. An alternative hypothesis is that these loci are the modifiers for other yet-to-be-identified mutations in the genome. Variability in these other factors, among individuals with the same microdeletion, may explain their varying phenotypes.

Although our results should be confirmed in other families carrying the same genomic microdeletions or duplications, we hope that this report would open a new genetic scenario leading to the search and possibly identification of genetic factors predisposing to MA disease, not only genic mutation, but also genome unbalances being candidate susceptibility factors.

## 5. Materials and Methods

Array CGH (aCGH) was performed on DNA from peripheral blood. Blood samples for DNA extraction were collected from the patient and her family, including parents, one brother, and one sister, after informed consent. Array-CGH analyses were performed using the oligo ISCA180K platform (BlueGnome array by Agilent Technologies, Santa Clara, CA, USA) according to the manufacturer’s protocol. Data were analyzed using Bluefuse software (BlueGnome, Cambridge, U.K.). Clinical interpretation of array CGH results were based on published literature and public databases such as ENSEMBL (www.ensambl.org), UCSC (genome.uscs.edu), Database for Genetic Variants (dgv.tcag.ca), DECIPHER (decipher.sanger.ac.uk) and the Italian database of Troina, (gvarianti.homelinux.net) following Cytogenetic European and International Guidelines [45,46]. Total RNA was isolated from lymphocytes of peripheral blood (from 3 independent blood collections) of the patient’s family (parents, one brother, and one sister) and 3 unrelated healthy controls (3 age- and sex-matched to the proband and with normal CGH analysis), using the RNeasy^®^ Mini Kit (Qiagen, Hilden, Germany, UE). mRNA expressions of *MC4R*, *MIR122*, and *RNF152* were studied by real-time PCR with SYBR green standard procedures (Applied Biosystem, Foster City, CA, USA), using the CFX96 system (Bio-Rad, Hercules, CA, USA) and analyzed by Bio-Rad CFX Manager software (www.bio-rad.com/en-eh/product/cfx-manager-software?ID=aed9803-cb4d-4ecb-9263-3efc9e650edb). Specific target sequences were selected for real-time PCR using Primer 3 software (http://frodo.wi.mit.edu/primer3/), analyzing two regions for each gene. The sequences of primers used (Metabion, International AG) were as follows: *MC4R* (forward 5′-CCAGTGAGTCCCTTGGAAAA, reverse 5′-CTTGGCTATTGCCACAATCA; forward 5′-CCCCATTCTTCCTCCACTTA, reverse 5′-TCCGGAGTGCATAAATCAGA), *mir122* (forward 5′-CTGAAAGGCCATAGCAGGAG, reverse 5′-CTGCCACCACTGTCAACAAG; forward 5′-GTAGGTGCCCTTCGGTACAA, reverse 5′-CTTTCTGCCCAGGAACTGTC) and *RNF152* (forward 5′-TCTCCAGCCCTAGAAGTTGC, reverse 5′-CCTTCACAGCGCTCTCTAATC; forward 5′, AGCACACCTGCTGTTCAGTG, reverse 5′-CTTCTGGCTGGTCCTCATCT). A control amplicon was selected with the same parameters in the *MAPK1* gene (forward 5′-CTAACGTTCTGCACCGTGAC, reverse 5′-GTGGTGTTGAGCAGCAGGTT) on 22q11.2; size (approximately 60 bp) and the melting temperature (60°C) were the same for all amplicons. Genomic coordinates herein are based on the February 2009 Human Genome Build (GRCh37/hg19).

The patient belongs to the MA cohort patients of the GENetics of mOyaMoyA (GENOMA) project, for which Ethical Committee approval was obtained (Regione Lombardia Ethical Committee, Fondazione Istituto di Ricovero e Cura a Carattere Scientifico IRCCS Neurologico Carlo Besta, n.12, October 1th, 2014). Specific consent was obtained from the patient and her relatives for clinical data, pictures, and DNA obtainment.

## Figures and Tables

**Table 1:** Sporadic syndromic cases of moyamoya angiopathy (MA).

Disease Name or Mutated Gene	Genes/Chromosomes	Clinical Features
*MA with autosomal dominant heritability*		
Type 1 Neurofibromatosis [12,13]	Neurofibromatosis 1 (*NF1*)	- Café au lait spots - Cutaneous/subcutaneous and/or plexiform neurofibroma - Optic pathway glioma - Lisch nodules - Skeletal abnormalities - Short stature - Possible systemic vasculopathy (aortic coarctation, renal artery stenosis, etc.)
Noonan Syndrome [14,15]	Protein Tyrosine Phosphatase, non-receptor Type 1 (*PTPN1*) (12q24.13), Son Of Sevenless homolog 1 (Drosophila) (*SOS1*) (2p22.1), v-Raf-1 murine leukemia viral oncogene homolog 1 (*RAF1*), (3p25.2); more rarely: v-Ki-ras2 Kirsten Rat Sarcoma viral oncogene homolog (*KRAS*) (12p12.1), Neuroblastoma RAS viral (v-ras) oncogene homolog (*NRAS*) (1p13.2), v-raf murine sarcoma viral oncogene homolog B1 (*BRAF*) (7q34), Mitogen-Activated Protein Kinase kinase 1 (*MAP2K1*) (15q22.31)	- Developmental delay and learning difficulties - Dysmorphisms (broad or webbed neck, unusual chest shape, low set nipple, facial dysmorphism) - Short stature - Congenital heart defect (pulmonary valve stenosis, hypertrophic cardiomyopathy, secundum atrial septal defect, etc.) - Ocular abnormalities - Auditory deficits - Cutaneous abnormalities (café au lait macules, lentigines) - Cerebrovascular abnormalities (aneurisms, arteriovenous malformations, etc.) - Cryptorchidism, male infertility
Costello Syndrome [16]	v-Ha-ras Harvey rat sarcoma viral oncogene homolog (*HRAS*) (11p15.5)	*Prenatal features:* prematurity, lymphatic dysplasia, macrosomia, fetal arrhythmias, etc. *Neonatal period:* - Severe feeding difficulties - Hypotonia, - Craniofacial dysmorphism (i.e., prominent epicanthal folds, full nasal tip, fleshy ear lobes) - Cardiac abnormalities (supraventricular tachycardias, hypertrophy, pulmonary valve stenosis, etc.) - Dermatological anomalies (cutis laxa, curly hair, acanthosis nigricans, papillomas, and loose thick skin on the dorsum of the hands and feet) - Developmental delay - Malignant tumor predisposition
Alagille Syndrome [17]	Jagged 1 (*JAG 1*) (20p12.2), *NOTCH2* (1p12-p11)	*Frequent symptoms:* - Cholestasis - Congenital heart disease (pulmonary artery stenosis) - Ophthalmologic abnormalities (commonly posterior embryotoxon) *Less frequent symptoms:* - Dysmorphic face (prominent forehead, deep-set eyes with moderate hypertelorism, pointed chin, and straight nose with a bulbous tip) - Systemic angiopathy (aortic coarctation, aneurysms, renal artery stenosis, etc.) - Skeletal abnormalities (commonly butterfly vertebrae). - Renal anomalies - Growth retardation
Marfan Syndrome [18]	Fibrillin 1 (*FBN1*)	- Skeletal manifestations (tall stature, pectus excavatum, or carinatum, higharched palate, arachnodactyly, laxity of ligaments with scoliosis, or joint hyperextensibility) - Ocular manifestations (strabismus, amblyopia, ectopia lentis, and cataract) - Cardiovascular manifestations (dissection or dilatation of ascending aorta, mitral valve prolapse, and cardiac electrical disturbances) - Cerebrovascular abnormalities (Intracranial aneurysms and spontaneous dissection of carotid. and vertebral arteries) - Nervous system involvement: dural ectasia, degenerative disc disease
*MA with autosomal recessive heritability*		
Sickle Cell Disease [19,20]	Hemoglobin Beta (*HBB*) (11p15.5)	- Vaso-occlusive events (i.e., dactylitis-pain and/or swelling of the hands or feet) - Spleen infarction - Chronic hemolytic anemia (anemia, jaundice, cholelithiasis) - Delayed growth and sexual maturation - Pulmonary artery hypertension - Priapism - Increased risk of bacterial infections
GUCY1A3 [11]	Guanylate Cyclase 1, soluble, Alpha 3 (*GUCYIA3*) (4q32.1)	- Severe achalasia - Arterial hypertension - Raynaud syndrome - Livedo reticularis - Erectile dysfunction
SAMHDI [21,22]	SAM domain and HD domain 1 (*SAMHD1*) (20q11.3)	*Aicardi-Goutières syndrome*: - Developmental and speech delay; intellectual disability - Truncal hypotonia, seizures, dystonia, microcephaly - Visual inattention, nystagmus, and abnormal eye movements - Skin manifestations (chilblain-like lesions, acrocyanosis, periungual erythema, or necrotic lesions of the toes, fingers, and outer helix) *Other symptoms in SAMHD1 mutations:* Congenital glaucoma, arthritis, chilblain lupus
Microcephalic Osteodysplastic Primordial Dwarfism, Type II (MOPD II)/ Majewski Syndrome [23,24]	Pericentrin (*PCNT*) (21q22.3)	- Severe intrauterine growth retardation - Severe disproportionate microcephaly and short stature - Bone radiologic abnormalities (dysplasia with metaphyseal changes in the limbs; epiphyseal delay; progressive loose-jointedness with occasional dislocation or subluxation of the knees, radial heads, and hips - Unusual facial features (prominent nose, eyes that appear prominent in infancy and early childhood, dysplastic ears - High squeaky voice; - Abnormally, small, and often dysplastic or missing dentition - Mild mental retardation - Insulin resistance
Seckel Syndrome (microcephalic primordial dwarfism) [25]	Ataxia Telangiectasia and Rad3 related (*ATR*) (3q23), Retinoblastoma Binding Protein 8 (*RBBP8*) (18q11.2), Centromere Protein J (*CENPJ*) (13q12.12), Centrosomal Protein 152kDa (*CEP152*) (15q21.1), Centrosomal Protein 63Da (*CEP63*) (3q22.2), Ninein (GSK3B interacting protein) (*NIN*) (14q22.1)	- Intrauterine growth retardation - Proportionate short stature - Severe microcephaly - Intellectual disability (plus variable structural brain anomalies) - Typical facial appearance with a prominent and beaked nose, sloping forehead, and micrognathia
*Genomic disorders*		
6p25.3-p23 del/dup and 12q24.32-qter dup [26]	On the 6p region: Interferon Regulatory Factor 4 (*IRF4*) and other 51 Online Mendelian Inheritance in Man (OMIM) genes including forkhead box C1 (*FOXC1*); on the 12q region: 22 OMIM genes not associated with genetic disorders.	- Hearing loss - Developmental delay/intellectual disability - Dysmorphic facies
Xq28 deletion [10,27]	Factor 8 (*F8*) (exons 1–6), FUN14 Domain Containing 2 (*FUNDC2*), mature T-cell proliferation 1 (*MTCP1*), nuclear gene encoding mitochondrial protein (*MTCP1NB*), BRCA1/BRCA2-containing complex, subunit 3 (*BRCC3*)	*Frequent symptoms:* - Short stature - Facial dysmorphism (hypertelorism, long philtrum, mild ptosis) - Hypergonadotropic hypogonadism Developmental delay, behavioral problems - Premature hair graying *Less frequent symptoms:* - Dilated myocardiopathy - Arterial hypertension - Osteopenia - Early onset cataract - Premature coronary heart disease
Smith-Magenis Syndrome (del 17p11.2-p13) [28,29]	More than 25 genes including: Retinoic Acid Induced 1 (*RAI1*), Mediator complex subunit 9 (*MED9*), RAS, dexamethasone-induced 1 (*RASD1*), Folliculin (*FLCN*), Peripheral Myelin Protein 22 (*PMP22*), Cytochrome C Oxidase assembly homolog 10 (*COX10*), ElaC ribonuclease Z 2 (*ELAC2*), Zinc Finger protein 18 (*ZNF18*), Myosin, Heavy chain 1 (*MYH1*)	- Dysmorphisms: brachycephaly, midface hypoplasia, prognathism, upslanting palpebral fissures, deep-set eyes, short-tipped nose, downturned corners of the mouth - Hoarse voice - Speech delay with or without hearing loss, psychomotor and growth retardation, and behavior problems - cardiac and renal anomalies - ophthalmologic disturbances (iris anomalies, microcornea, myopia, strabismus)
Trisomy 12p [30]	Genes included in the region of the rearrangement: 46, XX, rec(12)dup(12p)inv(12)(p11.2q24.3)mat	- Severe growth retardation - Developmental delay - Craniofacial dysmorphism (turricephaly, flat occiput, epicanthus, broad and long philtrum, everted lower lip, protruding tongue, tooth irregularity, small chin, flat nose, low-set ears, and abnormally-folded helix of ear - Limbs and truncal dysmorphic features short neck and abnormally-placed nipples; short and broad hands/feet, clinodactyly of the fifth finger, and foot deformities - Severe mental retardation. - Obesity and hypotonia in infancy - Central nervous system abnormalities (moderate ventricular dilatation; enlargement of Sylvian fissures, cortical sulci, and cisterna magna; hemispheric atrophy with microgyria; internal hydrocephalus; cortical dysplasia; ectopic glial tissue in the leptomeninges; and bilateral small basal ganglia) - Seizures
1p32p31 Deletion [31]	OMIM genes included in the region of the rearrangement: Complement component 8, Alpha polypeptide (*C8A*), Complement component 8, alpha polypeptide (*C8B*), Tumor-Associated Calcium Signal Transducer 2 (*TACSTD2*), Angiopoietin-like 3 (*ANGPTL3*), Forkhead box D3 (*FOXD3*), ALG6, alpha-1,3-glucosyltransferase (*ALG6*), Phosphoglucomutase 1 (*PGM1*)	- Developmental delay - Facial dysmorphisms - Brain malformations (absence or hypoplasia of the corpus callosum, ventriculomegaly, and macrocephaly) - Urinary tract defects (vesicoureteral reflux, urinary incontinence)
5q13.3 Duplication and 18 (Decipher ID: 263336)	Cholinergic Receptor, Nicotinic, alpha 7 (*CHRNA7*), Ovarian Tumor (OTU) domain containing 7A (*OTUD7A*) (our report)	- Facial dysmorphism - Overweight-obesity - Short stature - Liver steatosis
*Chromosome disorders*		
Down Syndrome [32]	21	- Developmental delay/intellectual disability - Facial dysmorphism - Congenital heart disease, vascular malformations, and traditional cardiovascular risk factors - Short stature - Thyroid disease - Cervical spine diseases - Hearing impairment - Overweight-obesity - Sleep apnea - Osteopenia
Turner Syndrome [33]	X	- Infertility - Aortic dilatation and dissections - Congenital heart defects and cardiac diseases - Short stature - Skeletal disproportion and dysmorphisms (micrognathia, higharched palate, short fourth metacarpals, genu valgum, Madelung wrist deformities, short limbs) - Mild cognitive deficits (visual spatial skills, processing visual cues, social difficulties, executive functions) - Metabolic syndrome and diabetes - Hepatic dysfunction - Hypertension - Autoimmune disorders
