# Correction: Sciacca, F. L., et al. Microduplication of 15q13.3 and Microdeletion of 18q21.32 in a Patient with Moyamoya Syndrome. *Int. J. Mol. Sci.* 2018, *19*, 3675

**DOI:** 10.3390/ijms21010020

**Published:** 2019-12-18

**Authors:** Francesca Luisa Sciacca, Ambra Rizzo, Gloria Bedini, Fioravante Capone, Vincenzo Di Lazzaro, Sara Nava, Francesco Acerbi, Davide Rossi Sebastiano, Simona Binelli, Giuseppe Faragò, Andrea Gioppo, Marina Grisoli, Maria Grazia Bruzzone, Paolo Ferroli, Chiara Pantaleoni, Luigi Caputi, Jesus Vela Gomez, Eugenio Agostino Parati, Anna Bersano

**Affiliations:** 1 Dipartimento di Diagnostica e Tecnologia Applicata, Fondazione IRCCS Istituto Neurologico Carlo Besta, 20133 Milan, Italy; francesca.sciacca@istituto-besta.it (F.L.S.); ambra.rizzo@istituto-besta.it (A.R.); 2 Laboratory of Cellular Neurobiology, Fondazione IRCCS Istituto Neurologico Carlo Besta, 20133 Milan, Italy; gloriabedini85@gmail.com (G.B.); sara.nava@istituto-besta.it (S.N.); 3 Unit of Neurology, Neurophysiology, Neurobiology, Department of Medicine, Università Campus Bio-Medico di Roma, 00128 Rome, Italy; f.capone@unicampus.it (F.C.); v.dilazzaro@unicampus.it (V.D.L.); 4 Neurosurgical Unit, Fondazione IRCCS Istituto Neurologico Carlo Besta, 20133 Milan, Italy; francesco.acerbi@istituto-besta.it (F.A.); paolo.ferroli@istituto-besta.it (P.F.); 5 Neurophysiopathology Department and Epilepsy Centre, Fondazione IRCCS Istituto Neurologico Carlo Besta, 20133 Milan, Italy; davide.rossi@istituto-besta.it (D.R.S.); simona.binelli@istituto-besta.it (S.B.); 6 Interventional Neuroradiology Unit, Fondazione IRCCS Istituto Neurologico Carlo Besta, 20133 Milan, Italy; giuseppe.farago@istituto-besta.it (G.F.); andrea.gioppo@istituto-besta.it (A.G.); 7 Neuroradiological Unit, Fondazione IRCCS Istituto Neurologico Carlo Besta, 20133 Milan, Italy; marina.grisoli@istituto-besta.it (M.G.); maria.bruzzone@istituto-besta.it (M.G.B.); 8 Developmental Neurology Division, Fondazione IRCCS Istituto Neurologico Carlo Besta, 20133 Milan, Italy; chiara.pantaleoni@istituto-besta.it; 9 Cerebrovascular Unit, Fondazione IRCCS Istituto Neurologico Carlo Besta, 20133 Milan, Italy; luigi.caputi@istituto-besta.it (L.C.); Jesus.VelaGomez@istituto-besta.it (J.V.G.); eugenio.parati@istituto-besta.it (E.A.P.)

The authors wish to make the corrections to this paper [1]. The surnames and names of all authors were switched in the original paper; the following being the right sequence: Francesca Luisa Sciacca, Ambra Rizzo, Gloria Bedini, Fioravante Capone, Vincenzo Di Lazzaro, Sara Nava, Francesco Acerbi, Davide Rossi Sebastiano, Simona Binelli, Giuseppe Faragò, Andrea Gioppo, Marina Grisoli, Maria Grazia Bruzzone, Paolo Ferroli, Chiara Pantaleoni, Luigi Caputi, Jesus Vela Gomez, Eugenio Agostino Parati and Anna Bersano.

The authors would like to apologize for any inconvenience caused to the readers by these changes.

